# Structural Insight Into the Role of Mutual Polymorphism and Conservatism in the Contact Zone of the NFR5–K1 Heterodimer With the Nod Factor

**DOI:** 10.3389/fpls.2018.00344

**Published:** 2018-04-11

**Authors:** A. A. Igolkina, Yu B. Porozov, E. P. Chizhevskaya, E. E. Andronov

**Affiliations:** ^1^Laboratory of Microbiological Monitoring and Bioremediation of Soil, All-Russia Research Institute for Agricultural Microbiology, St. Petersburg, Russia; ^2^Mathematical Biology and Bioinformatics Laboratory, Institute of Applied Mathematics and Mechanics, Peter the Great St. Petersburg Polytechnic University, St. Petersburg, Russia; ^3^The Laboratory of Bioinformatics, ITMO University, St. Petersburg, Russia; ^4^The Laboratory of Bioinformatics, I.M. Sechenov First Moscow State Medical University, Moscow, Russia; ^5^Laboratory of Biology and Biochemistry of Soils, VV Dokuchaev Soil Science Institute, Moscow, Russia; ^6^Saint Petersburg State University, St. Petersburg, Russia

**Keywords:** NFR5, K1, Nod factor, heterodimeric receptor, population polymorphism, sliding cylinder, mutual polymorphism

## Abstract

Sandwich-like docking configurations of the heterodimeric complex of NFR5 and K1 *Vicia sativa* receptor-like kinases together with the putative ligand, Nod factor (NF) of *Rhizobium leguminosarum* bv. *viciae*, were modeled and two of the most probable configurations were assessed through the analysis of the mutual polymorphisms and conservatism. We carried out this analysis based on the hypothesis that in a contact zone of two docked components (proteins or ligands) the population polymorphism or conservatism is mutual, i.e., the variation in one component has a reflected variation in the other component. The population material of 30 wild-growing *V. sativa* (leaf pieces) was collected from a large field (uncultivated for the past 25-years) and pooled; form this pool, 100 randomly selected cloned fragments of *NFR5* gene and 100 of *K1* gene were sequenced by the Sanger method. Congruence between population trees of *NFR5* and *K1* haplotypes allowed us to select two respective haplotypes, build their 3D structures, and perform protein–protein docking. In a separate simulation, the protein-ligand docking between NFR5 and NF was carried out. We merged the results of the two docking experiments and extracted NFR5–NF–K1 complexes, in which NF was located within the cavity between two receptors. Molecular dynamics simulations indicated two out of six complexes as stable. Regions of mutual polymorphism in the contact zone of one complex overlapped with known NF structural variations produced by *R. leguminosarum* bv. *viciae*. A total of 74% of the contact zone of another complex contained mutually polymorphic and conservative areas. Common traits of the obtained two stable structures allowed us to hypothesize the functional role of three-domain structure of plant LysM-RLKs in their heteromers.

## Introduction

Lysin motif receptor-like kinases (LysM-RLKs) are one of the most diverse and abundant families of plant membrane receptors responsible for specific recognition of biotic and abiotic signals (Tax and Kemmerling, 2012; Zipfel and Oldroyd, 2017). The precise evolutionary history of LysM-RLKs is not known, however, it inevitably includes gene duplications, rearrangements and neofunctionalization, resulting in a broad spectrum of plant receptors specialized to recognize particular molecules (De Mita et al., 2006; Zhang et al., 2007, 2009; Fliegmann and Bono, 2015). While some of these receptors are tuned to recognize primitive signals from pathogens, such as chitin and chitosan of fungal cell walls, others provide specificity for highly decorated signaling molecules like Nod and Myc factors that initiate the plant–bacteria or arbuscular mycorrhizal symbioses. The evolutional transformation from defense to the symbiosis is accompanied by increased specificity for molecular signals: defense works against many fungi species, whereas the symbiosis is established with a limited range of bacterial or fungal species.

One example of the high specificity exhibited by the system of LysM-RLKs toward signaling molecules is the nitrogen-fixing rhizobia-legume symbiosis (Wang et al., 2012). Containing extracellular LysM domains, some legume LysM-RLKs can perceive a special group of bacterial signaling molecules, Nod factors (NFs), and then subsequently activate of the intracellular kinase domain that triggers downstream signal transduction and initiates formation of the symbiosis. Phylogenetic analysis of LysM-RLKs defined two subfamilies: LYK and LYR. Some of their members—NFR1 and NFR5—were considered as NF receptors. NFR1 (LjNFR1/MtLYK3/PsSym37) carries an active protein kinase domain whereas NFR5 (LjNFR5/MtNFP/MtLYR3/PsSym10) does not contain an activation loop in the kinase domain. In order to be functionally significant, NFR5 was proposed to be a subunit in a heterodimer with NFR1 (Madsen et al., 2003; Radutoiu et al., 2003; Arrighi et al., 2006), and this hypothesis was subsequently confirmed. To be specific, the physical possibility of dimerization between MtLYK3 and MtLYR3 and between PsSym10 and PsK1 was shown (Fliegmann et al., 2016; Kirienko et al., 2017). Recent studies have proposed that receptor complexes recognizing NFs and transmitting the signal intracellularly can be also heterotrimeric (Oldroyd, 2013; Zipfel and Oldroyd, 2017).

Neither precise NF binding site in the receptors (NFR5 and NFR1) nor a contact zone in the NFR1-NFR5 heterodimer has been already identified. While the second issue was not intensively addressed, there are many studies investigating the first one. Analyses of mutants of NFR1 and NFR5 homologs revealed several mutations that are essential for symbiosis formation and, therefore, probably located in close proximity to the NF binding site. Six essential positions were found in NFR1 and NFR5 homologs: the Leu77 amino acid position in the first of three LysM domains (LysM1) of PsSYM37 (Zhukov et al., 2008); the Pro169 and Ser59 positions in PsK1 (Kirienko et al., 2017); the Leu118 position in LysM2 of LjNFR5 (Radutoiu et al., 2007); the Leu154 position in LysM2 of MtNFP (Bensmihen et al., 2011); and the Tyr228 position in LysM3 of MtLYR3 (Malkov et al., 2016). Most of these positions are located around a shallow groove on the LysM domains, which is a standard pose of ligands in crystal structures of close homologs to LysM-RLKs and other LysM-containing proteins in a range of organisms such as *Arabidopsis thaliana* AtCERK1 (PDB codes: 4EBZ), rice *Oryza sativa* OsCEBiP (PDB code: 5JCE), fungal pathogen *Passalora fulva* PfEcp6 (PDB code: 4B8V) and proteins of *Magnaporthe oryzae* (PDB codes: 5C8P and 5C8Q) as well as the bacterium *Thermus thermophiles* (PDB code: 4UZ3). Despite these pieces of evidence that do not contradict the hypotheses that the NF binding site is located in the shallow groove on a LysM domain, the precise binding site in NFR1 and NFR5 has not determined yet.

All of the attempts to model the interaction between NF and LysM-RLKs tried to bind the signaling molecule into the standard shallow groove on a LysM surface (Mulder et al., 2006; Fliegmann et al., 2013; Hayafune et al., 2014; Mesnage et al., 2014; Malkov et al., 2016) These studies utilized a single LysM domain (out of three) detached from the receptor structure and none of the studies employed docking of the NF with the dimer of NFR1 and NFR5 homologs. The previous studies thus state the principal possibility to dock the NF's chitin backbone into the shallow groove near to the abovementioned essential positions. However, the functional role of both the three-LysM-domain structure of the receptor as well as the receptor-receptor dimer is still not revealed by the molecular modeling.

One aim of our study was to shed the light on a possible configuration of heterodimeric receptors with bound NF, while the second was to interpret the functional role of population polymorphism in context of the NF–receptor complex. This study is related to the recently introduced hypothesis, the “Evolutionary Moulding” hypothesis (Igolkina et al., 2018), which implies the interaction between legume and rhizobial populations at the genetic level. In particular, it was demonstrated that the population diversity of the *V. sativa NFR5* gene (homolog of *NFR5*) was matched with the diversity of *nodA* gene pool extracted from symbiotic root nodule population of *R. leguminosarum bv. viciae* (Igolkina et al., 2018). It was proposed that the observed relationship was mediated by the population variation in NF decorations, which are small chemical substituents (like O-acetyl, O-sulfyl, O-fucosyl, or minor modifications of the unsaturated fatty acid chain) modifying the conservative core part of NF (Spaink, 2000). In this study, we hypothesized the presence of the Evolutionary Moulding effect at the level of gene products, i.e., the variation in NF structure has a respective variability within its binding site on a receptor. We also considered that the analysis of population polymorphism on the surface of the receptor could suggest potential NF binding sites (Pazos et al., 1997; Bai et al., 2016; Rodriguez-Rivas et al., 2016).

Here we analyzed the population polymorphism data and 3D structures of two receptors—VsNFR5 (homolog of NFR5) and VsK1 (homolog of NFR1). A recent study demonstrated that the close homologous receptors PsSym10 and PsK1 in *P. sativum* formed the heterodimer (Kirienko et al., 2017); both of the receptors are expressed in roots (Zhukov et al., 2008). Therefore, we decided that the VsNFR5 and VsK1 receptors were good candidates to investigate the complex of VsNFR5–VsK1 heterodimer with the NF (NFR5–NF–K1).

As discussed below, we developed a method to investigate possible NFR5–NF–K1 complexes integrating the molecular modeling and population polymorphism analysis. We demonstrated the principal possibility of the NFR5–K1 heterodimer and the molecular binding of this complex with the NF of *R. leguminosarum* bv. *viciae*. Employing the population diversity in the receptor genes we selected the respective haplotypes of *NFR5* and *K1* genes for modeling and revealed putative NFR5–NF–K1 complexes exhibiting to the mutual polymorphism and conservatism in the contact zone of these complexes.

## Materials and methods

### Sampling and sequencing

Thirty of three wild growing plants with root nodules of the common vetch *V. sativa* were uniformly collected from a large fallow field near Vyritsa (Gatchinskii region of Leningradskaya oblast, Russia, 59°24′7.74″ N; 30°15′28.74″ E), uncultivated for 25 years. We pooled of leaf pieces (0.1 g per plant sample) for DNA extraction. We pooled 0.1 g of leaf tissue from each of the plant samples.

DNA from the pool was isolated by AxioPrep kit (Axigen) and was used as the template DNA for PCR amplifications. First, 837 bp DNA fragments of the plant receptor gene, *NFR5*, containing signal peptide, all three LysM domains and a part of transmembrane domain were amplified with the following pairs of primers: forward “nfr5-for4” (5′-AAGTCTTGGTTGTTACTTGCC-3′) and reverse “nfr5-Grev3” (5′-CACCTGAAAGTAACTTATCYGCA-3′). Second, 714 bp DNA fragments of the plant receptor gene, *K1*, containing signal peptide, all three LysM domains and a part of transmembrane domain were amplified with the following pairs of primers: forward “k1-for” (5′-GCTCTCTTTCTTATTGACCAAA-3′) and reverse “k1-rev” (5′-CACCTGAAAGTAACTTATCYGCA-3′). The standard PCR protocol used consisted of initial denaturation at 95°C for 3 min, 30 cycles with denaturation at 94°C for 30 s, primer annealing at 48°C for 30 s, extension at 72°C for 1 min and final extension for 4 min. PCR fragments were extracted from agarose gel (Onishchuk et al., 2015) and cloned into the plasmid pTZ57R/T (Thermo Scientific, Lithuania).

Next, 100 randomly selected cloned fragments of each gene—*NFR5* and *K1*—were sequenced by the Sanger method in an automated ABI 3500xL sequencer (Applied Biosystems) using standard M13 (−20) and (−26) primers. GenBank accession numbers for *NFR5* and *K1* sequences are as follows: Popset ID 1041522217 and Nucleotide database IDs MF692841-MF692940, respectively. The multiple alignment of 100 sequences was performed with ClustalW as implemented in MEGA6 (Tamura et al., 2013).

### Congruence of *NFR5* and *K1* populations gene trees

For each set of *NFR5* and *K1* gene sequences from the *V. sativa* population we took unique haplotypes, counted their frequencies and then collapsed them to 15 pseudo-haplotypes, as it was described in Igolkina et al. (2018). Then, each population was represented as a Gaussian Mixture Model (GMM) in three-dimensional Euclidean space. Procrustes analysis, employing rotation, translation and mirror reflection, was performed to match GMMs corresponding to *NFR5* and *K1* genes. The concordance between GMMs was assessed by Δ*G* values. The lower a Δ*G* value is, the more similar two GMMs are, and consequently, the more similar the topologies of the two population gene trees. We set the null hypothesis that the obtained Δ*G* value is not lower than a Δ*G* value calculated for random frequencies of pseudo-haplotypes. To test this hypothesis, we performed shuffling on the frequencies 1,000 times.

For the visual comparison of plant and rhizobial populations, we constructed tanglegrams based on adjusted GMMs after Procrustes superimposition. A tanglegram is a diagram with a pair of two binary trees with matching leaves connected by edges. To construct them, we built two NJ gene trees for 15 *NFR5* pseudo-haplotypes and 15 *K1* pseudo-haplotypes and plotted the trees face to face. A pair of leaves from the different trees was connected by an edge if a point in the Euclidean space corresponding to one leaf was located within the five closest points to a point of another leaf and vice versa (Igolkina et al., 2018).

### Homology modeling of NFR5 and K1 receptors

Multiple alignment of 50 unique amino acid sequences of the *V. sativa NFR5* gene was performed with the ClustalX program (Larkin et al., 2007); the number of unique nucleotide sequences was 77. The homology modeling server SWISS-MODEL (Biasini et al., 2014) was used to build the domain models for 50 target sequences. For all queries, the server defined three top templates: *Arabidopsis* chitin receptor kinase AtCERK1 (PDB code: 4EBZ), Rice Chitin Receptor OsCEBiP (PDB code: 5JCE) and sugar binding protein of *P. fulva* (PDB code: 4B8V). We worked with models built on the first template, as it was the one with the highest sequence identity (23% of sequence identity on average and 180 bp target coverage). The same pipeline was performed for the set of sequences of K1 gene products (44% of sequence identity on average and 190 bp target coverage). The number of unique nucleotide sequences was 36 and the number of unique translated sequences was 30. A total of 30 models of the K1 receptor were built by SWISS-MODEL server based on the AtCERK1 template (PDB code: 4EBZ).

Each model structure was prepared by Protein Preparation Wizard in Schrödinger 2016-3 software following its Protein Preparation guideline (Sastry et al., 2013). The structures were refined by minimization subjected to the OPLS2005 force field and loop geometries optimization in the Schrödinger Prime package. Model quality was assessed using a Ramachandran plot and Protein Reports. Molecular surfaces of the medium resolution were created using the 1.0 scaling factor for the van der Waals radius and a rolled probe radius of 1.4 Å. Assessment of NFR5 protein active sites was conducted by the Schrödinger SiteMap application (Halgren, 2007). Based on the collection of NFR5 models, we assessed the matrix of frequencies that two amino acids placed in one active site, {*M*_*i,j*_}:

$$\begin{array}{l} M_{i,j}=\overset{N_{m}}{\underset{m=1}{\sum }}f_{m}\overset{N_{m,s}}{\underset{s=1}{\sum }}I_{m,s}{\left(i\right)}^{*}I_{m,s}\left(j\right), \end{array}$$

where *N*_*m*_ is the number of modeled haplotypes, *f*_*m*_ is the frequency of *m*th haplotype in population, *N*_*m,s*_ is the number of active sites predicted for *m*th model, and *I*_*m,s*_(*i*) is an indicator function that indicates membership of an *i*th amino acid in the *s*th active site of *m*th model. We then biclustered the matrix in order to reveal groups of amino acids that represented the most probable binding sites.

### Ligand preparation

The initial flat structure of the NF ligand was created in the 2D Sketcher module in Schrödinger 2016-3 (Supplementary Figure S1). The NF structure represented the most abundant variant synthesized by *R. leguminosarum* bv. *viciae* population. The chitin backbone contained four N-acetylglucosamine residues and the NF modifications were the same as produced by RBL5560 strain (Spaink, 2000). The collection of 10 low-energy 3D ligand structures was generated at the neutral pH and under the OPLS2005 force field by the Schrödinger LigPrep module.

### Docking

The receptor grid was generated in six putative regions (LysM1/2/3 domains and three grooves between the domains) at the determined centroids (see Supplementary Table S1) using the 1.0 scaling factor for the van der Waals radius in the Glide application (Friesner et al., 2006). Characteristic sizes of the midpoint box and enclosing box were 14 and 23 Å, respectively. Ligand docking was performed in the Glide application using the extra precision mode (XP) and the flexible ligand sampling with fixed ring conformation. Ligand-receptor interaction energy was estimated by the MM-GBSA approach in Prime module of Schrödinger.

The protein–protein docking was performed in the Schrödinger BioLuminate module (Zhu et al., 2014) searching for the 30 best complexes within 70,000 (the default numbers) possible protein–protein configurations without constraints. The docking was performed as rigid body optimization with no subsequent minimization of the interfacial region (Beard et al., 2013).

### Sliding cylinder for the analysis of responding polymorphism and conservatism

We developed a method to analyse the responding polymorphism and conservatism in a contact zone of a dimer complex. The method consisted of two steps: (1) constructing the contact zone surface and (2) mapping the population polymorphism from each subunit to the surface.

**Figure 1:**
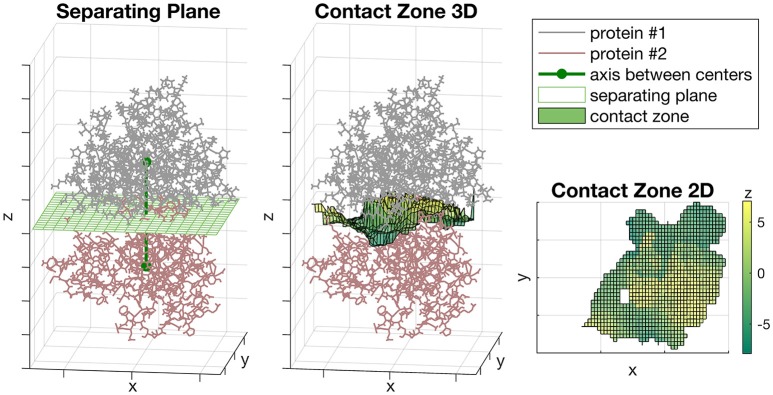
The Sliding cylinder technique. (**Left**) The separating plane with a grid is constructed between the proteins. (**Middle**) The contact zone is a transformation of the plane which curves aspiring to a better isolation of proteins. (**Right**) The landscape of the contact zone. The unit of measurement for each axis is Å, however, they were omitted as being non-informative here.

We rotated the complex so the axis passing through the centers of proteins was parallel to the Z-axis (Figure 1, left). The zero position of Z-axis was set to the midpoint between centers of proteins. The unit of measurement for each axis was Å. After that, we constructed the separating plane parallel to XoY coordinate plane passing through the zero position of Z-axis and define the orthogonal grid on it (Figure 1, left). Then, for each grid node (*x*_*i*_, *y*_*i*_) we defined the *z*_*i*_ value equal to the midpoint between two proteins on the axis passing through the (*x*_*i*_, *y*_*i*_) and parallel to Z-axis (Figure 1, middle). If the distance between two proteins in this vertical direction was more than a predefined threshold, then *z*_*i*_ was not considered. The resultant set of points {(*x*_*i*_, *y*_*i*_, *z*_*i*_)}, where *z*_*i*_ was defined, formed the shape of the contact zone between subunits. The possible shape of a contact zone is shown in (Figure 1, right), where the color denotes the *z*_*i*_ coordinate.

At the second step, we used the Sliding cylinder technique to create the projection of the diversity of each subunit protein to grid nodes of the contact zone, described as follows. Set as fixed the location of one subunit protein. Let *P*^*C*^ be a set of protein atoms located inside a cylinder which center is located at a grid point *C*, the axis is parallel to Z-axis and the radius equal to *r*. Let a set of atoms *P*^*C*^ belong to the respective set of protein residues $R^{C}={\left\{R_{i}\right\}}_{\;}$, where *R*_*i*_ denotes a residue. Let the residue *R*_*i*_ correspond to the set of atoms $P^{R_{i}}$. The portion of atoms of *R*_*i*_ inside the cylinder is $f_{i}=|P^{C}\cap P^{R_{i}}|/|P^{R_{i}}|.\;$Let the mean distance from a residue *R*_*i*_ to the cylinder center be $d_{i}=\frac{1}{\left|P^{R_{i}}\right|}\underset{P\in P^{R_{i}}}{\sum }||P,C||,$ and let the weight, *w*_*i*_, of the residue *R*_*i*_ reflect the Gauss decay score as follows:

$$\begin{array}{l} w_{i}=f_{i}\cdot \exp\left(-\frac{{\left(d_{i}-\underset{j,R_{j}\epsilon R^{c}}{\min}d_{j}\right)}^{2}}{h}\right), \end{array}$$

where *h* is a smoothing parameter or a bandwidth and here equals to *s*^2^/2, where *s* is a mean size of the subunit protein, i.e., the mean distance between protein atoms and the protein center.

Population amino acid diversity within the cylinder is calculated based on the multiple alignment of protein sequences shortened to amino acid positions corresponding to *R*^*C*^. Let this shortened alignment be presented by the matrix *A*:*N*_*seqs*_ × *N*_*res*_, where *N*_*seqs*_ is the number of protein sequences and $N_{res}=|R^{C}|$. Then the amino acid diversity within the cylinder is calculated as follows:

$$\begin{array}{l} \pi_{C}=\frac{2}{N_{seqs}\cdot \left(N_{seqs}-1\right)\cdot N_{res}}\overset{N_{seqs}}{\underset{\begin{array}{c} j,k=1\\ j>k \end{array}}{\sum }}\overset{N_{res}}{\underset{i=1}{\sum }}\left(1-I\left(A_{j,i},A_{k,i}\right)\right)\cdot w_{i}, \end{array}$$

where the first multiplier is the normalization factor; *I*(*x, y*) is the indicator function, $I\left(x,y\right)=\{\begin{array}{c} 1,if\;x=y\\ 0,\;if\;x\neq y \end{array}\;$.

The *dN*/*dS* ratio within the cylinder is calculated by analogy with the above. Let $k_{i}^{\left(n,sub\right)}$ and $k_{i}^{\left(s,\;sub\right)}$ be the numbers of non-synonymous and synonymous substitutions in the population within the *i*th amino acid site (*i*th residue). Let $k_{i}^{\left(n,site\right)}$ and $k_{i}^{\left(s,\;site\right)}$ be the total numbers of possible non-synonymous and synonymous substitutions within the *i*th site, which are estimated based on the multiple alignment of nucleotide sequences of the protein. Then the cylinder-based *dN*/*dS* is calculated as follows:

$$\begin{array}{l} \frac{dN}{dS}=\frac{\sum_{i=1}^{N_{res}}\left(k_{i}^{\left(n,sub\right)}\cdot w_{i}\right)/\sum_{i=1}^{N_{res}}\left(k_{i}^{\left(n,site\right)}\cdot w_{i}\right)}{\sum_{i=1}^{N_{res}}\left(k_{i}^{\left(s,\;sub\right)}\cdot w_{i}\right)/\sum_{i=1}^{N_{res}}\left(k_{i}^{\left(s,\;site\right)}\cdot w_{i}\right)} \end{array}$$

After the measure (π_*C*_ or *dN*/*dS*) is calculated within each grid node, the distribution of values across the contact zone is denoted as a map. In order to compare two maps from both sides of the contact zone (from both subunits), we constructed a so-called Red-Green (RG)-plot. In this plot, two maps are represented by the different color channels (red and green) and overlapped. The intensity of a color is higher for higher values. Then we applied a filter to distinguish four areas:

- Black-colored areas show a mutually conservative zone, where intensities of both red and green channels were <20% of the maximum intensity.
- Green-colored areas show a zone where the intensity of the green channel is more than twice as large as the intensity of the red channel.
- Red-colored areas show a zone where the intensity of the red channel is more than twice as large as the intensity of the green channel.
- Yellow-colored areas show a mutually polymorphic zone, where both receptors display a comparable level of polymorphism.

The introduced filter is presented in the **Figure 7**. Mutual (black and yellow) and one-channel (red and green) areas are equiprobable under the filter. In our study, we took the radius of the cylinder *r* = 4 and the grid step as to 1 for both the X and Y axes.

### Distance and overlap between the ligand and the receptor

Let the ligand *L* and the receptor *R* be defined by the positions of their atoms: $L:\;{\left\{L_{i}\right\}}_{i=\bar{1,N_{L}}}$; $R:{\left\{R_{j}\right\}}_{j=\bar{1,N_{R}}}$. Then the mean distance between *L* and *R* is defined as follows:

$$\begin{array}{l} d\left(L,\;R\right)=\frac{1}{N_{L}}\overset{N_{L}}{\underset{i=1}{\sum }}\underset{j=\bar{1,N_{R}}}{\min}|L_{i}R_{j}|. \end{array}$$

The minimum distance between *L* and *R* is calculated as:

$$\begin{array}{l} d_{m}\left(L,R\right)=\underset{\begin{array}{c} j=\bar{1,N_{L}}\\ j=\bar{1,N_{R}} \end{array}}{\min}|L_{i}R_{j}|. \end{array}$$

The number of overlapped atoms between *L* and *R*, *N*_*o*_(*L, R*), is calculated with respect to the overlapping threshold *t*_*o*_:

$$\begin{array}{l} N_{o}\left(L,\;R\right)=\overset{N_{L}}{\underset{i=1}{\sum }}\left(\underset{j=\bar{1,N_{R}}}{\min}|L_{i}R_{j}|<t_{o}\right). \end{array}$$

### Molecular dynamics

While the simultaneous docking of three structures—two proteins (NFR5 and K1) and one ligand (NF)—is not possible by Schrödinger tools, we construct the complex of interest in three steps. First, the NF was docked to the NFR5 receptor by proteins—ligand docking in the Glide application. Then, NFR5, and K1 were docked to each other by protein-protein docking in BioLuminate module (see Docking section). Last, two docking complexes were merged into one NFR5–K1–NF complex overlapping by NFR5. After that, we performed the Desmond molecular dynamics simulation (Shivakumar et al., 2010) to analyse the stability of each complex.

The system for molecular dynamics was built using the TIP3P water model, then passed minimization and 100 ns molecular dynamics simulation (NVT ensemble, *T* = 300 K). After the simulation, all of the frames in the simulation trajectory were grouped into five clusters based on RMSD values. The optimal complex was determined as representative of the greatest cluster. The optimal complex was then compared with the initial one. If these complexes were topologically similar, then we denoted the initial complex as stable.

## Results

### Phylogeny

**Figure 2:**
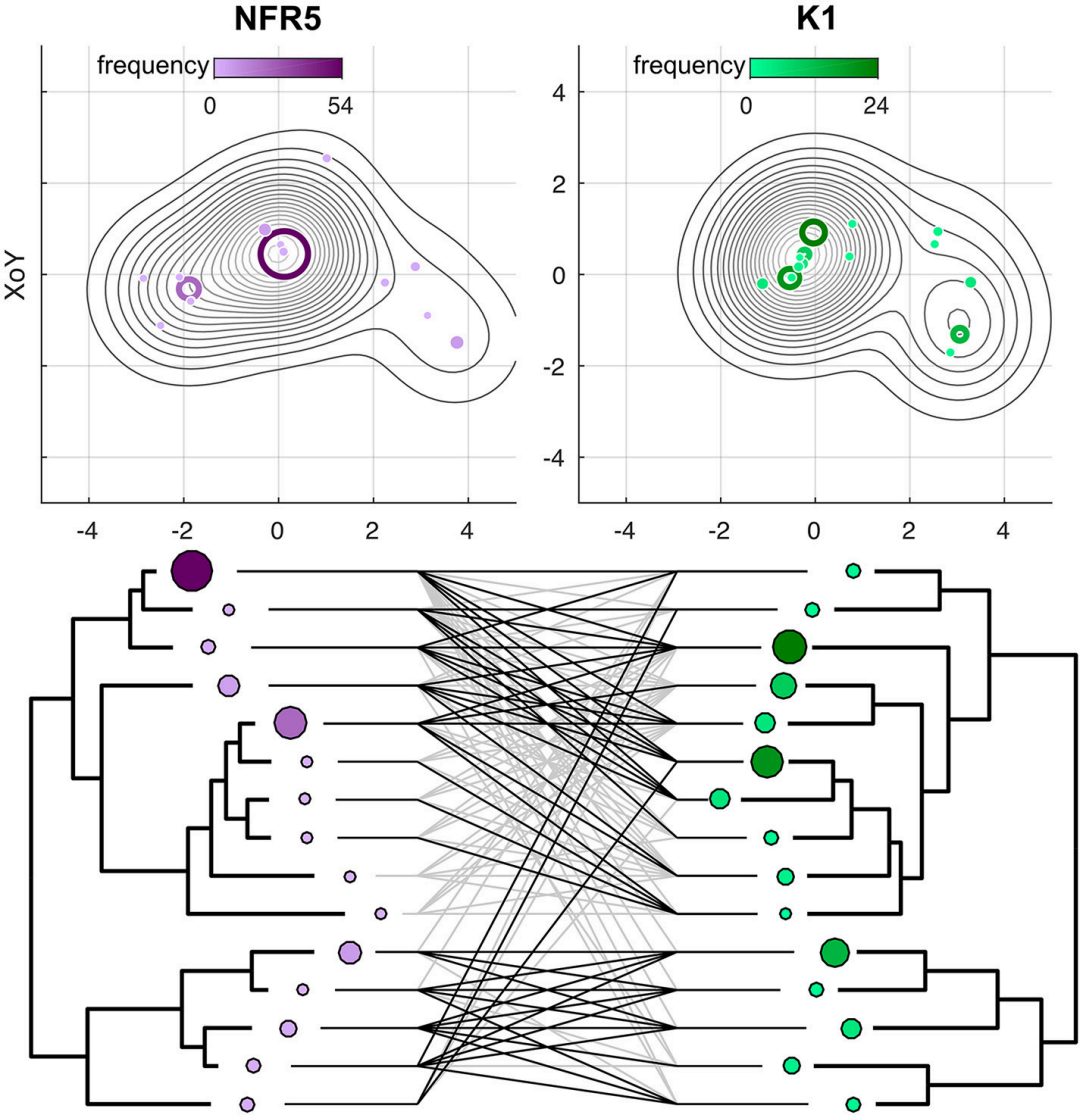
Projections of Gaussian Mixture Models (GMMs) of NFR5 and K1 population gene sets, respectively, to the XoZ coordinate plane. Tanglegrams were built on the distance matrices within NFR5 population gene set and within K1 population gene set after the Procrustes superimposition of GMMs.

We analyzed the correspondence in haplotypes between the *NFR5* and *K1* gene sets. A population set of 100 sequences for *NFR5* gene contained 50 unique amino acid haplotypes while the set of 100 sequences for *K1* gene contained 30 unique amino haplotypes. For each gene, haplotypes were grouped into 15 pseudo-haplotypes that were used to construct population trees for *NFR5* and *K1* genes. The difference between the trees was assessed by a *G* value, which was significantly lower (*p* < 0.05) than calculated for a random distribution of pseudo-haplotype frequencies across leaves of trees (Supplementary Figure S2). Hence, topologies of *NFR5* and *K1* gene trees were significantly similar, and the constructed tanglegram illustrated this congruence (Figure 2). This result allowed us not only to choose the corresponding dominant alleles of *NFR5* and *K1* genes for further docking but also to support the NFR5–K1 physical interaction (especially considering the fact that *V. sativa* is a cross-pollinated plant).

### Models

**Figure 3:**
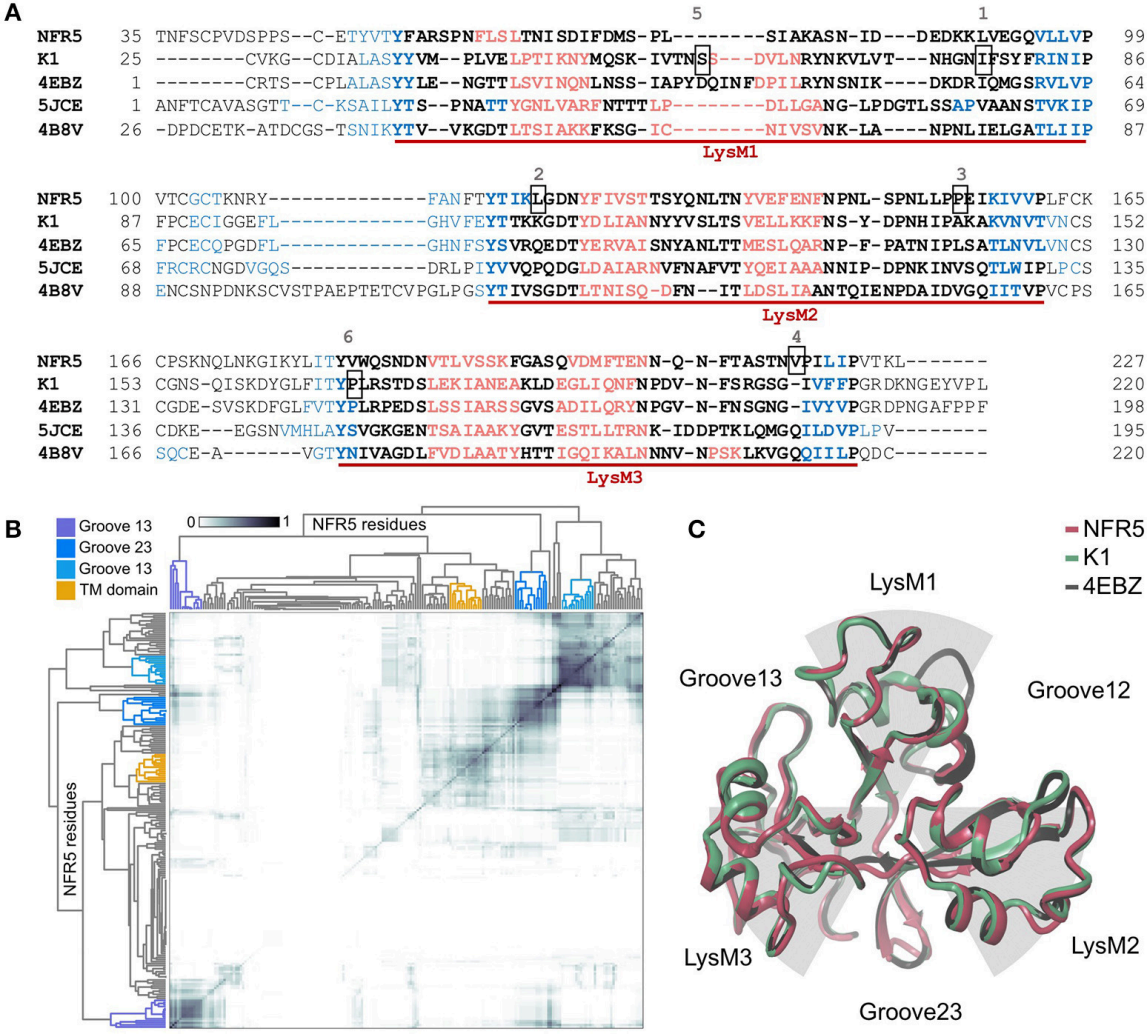
**(A)** Multiple Alignment of NFR5 and K1 gene sequences with three homologs, for which the crystal structure is known. Three LysM domains of the βααβ-fold motif are marked with red lines: alpha-helixes are colored in red, beta-strands are colored in blue. Six positions are squared: 1—homologous position for Leu77 in PsSYM37 (Zhukov et al., 2008); 2—homologous position for Leu118 in LjNFR5 (Radutoiu et al., 2007); 3—homologous position for Leu154 in MtNFP (Bensmihen et al., 2011); 4—homologous position for Tyr228 in MtLYR3 (Malkov et al., 2016); 5—homologous position for Ser59 in PsK1 (Kirienko et al., 2017); 6—homologous position for Pro169 in PsK1 (Kirienko et al., 2017). **(B)** Results of clustering of NFR5 residues to reveal the most probable active sites predicted by SiteMap, axes of the HeatMap represent all NFR5 residues. **(C)** Results of homology modeling of NFR5 and K1 receptors with their template [PDB ID: 4EBZ], up-view.

The 3D structures of 50 unique *NFR5* haplotypes and 30 unique *K1* haplotypes were built by SWISS-MODEL service (https://swissmodel.expasy.org/interactive). The modeled fragments represented the extracellular part of receptors and contained three LysM domains of the βααβ-fold motif: two alpha-helixes flanked by two beta-strands. The first LysM domain in the NFR5 models was slightly degenerated in the second alpha-helix (Figure 3A). Lengths of the modeled fragments were 195 and 198 amino acids for the NFR5 and K1 receptors, respectively.

For the collection of NFR5 models, we predicted the poses of active sites by the SiteMap tool in Schrödinger, clustered them across the collection, which resulted in four distinct clusters (Figure 3B). Three of them represented grooves between LysM domains and the fourth—the area connecting with the transmembrane domain. According to the *in silico* results, we considered three grooves between LysM domains (Figure 3C) as possible regions for NF binding.

### Polymorphism along the gene sequence and within six regions of receptors

**Figure 4:**
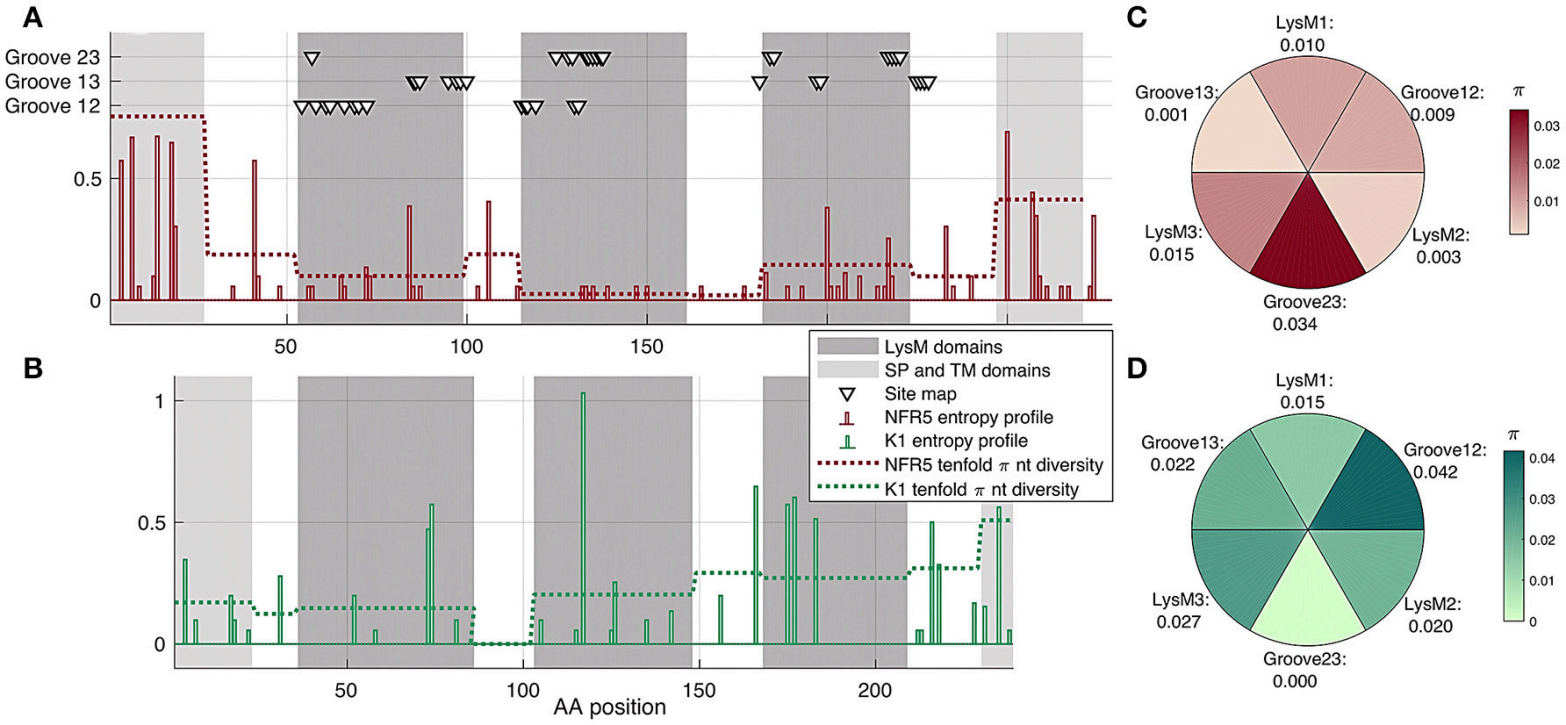
**(A,B)** Shann on entropy profiles (solid lines) and π diversity within domains (dashed lines) and interdomain regions of *NFR5* and *K1* receptor genes. Domains are highlighted in gray. Three clusters of residue positions, obtained by SiteMap analysis, are marked with triangles **(C,D)** π diversity within six putative regions for NF binding on NFR5 and K1 receptors, up-view.

We analyzed the population polymorphism within *NFR5* and *K1* receptor gene sets as polymorphism within linear gene fragments and within neighboring 3D regions. The population amino acid diversity within five domains (SP, LysM1/2/3, TM) and inter-domain regions of *NFR5* and *K1* receptor genes was estimated (Figures 4A,B). The Shannon's entropy profile was calculated within each amino acid position to indicate the most variable positions. The polymorphism profile for the *NFR5* gene had increased variability in the flanking domains (SP and TM) and the lowest polymorphism in the LysM2 domain (Figure 4A). The polymorphism profile for *K1* gene contained an almost-conservative inter-domain region between LysM1 and LysM2 and the moderate variability in other regions (Figure 4B).

Together with the “linear” polymorphism, we calculated the amino acid diversity within six parts of receptor surfaces: three LysM domains and three grooves between the domains. The grooves on the NFR5 receptor contained the active sites predicted by the SiteMap and did not represent any line segment of the protein sequence (triangles in Figure 4A). We found that the groove between LysM2 and LysM3 (Groove23) in NFR5 receptor was the most polymorphic (Figure 4C) and, at the same time, the most spacious one. In contrast to the diversity within the inter-domain region between LysM1 and LysM2 of K1, the Groove12 was found as the most variable, while the most conservative one was Groove23 (Figure 4D).

The results indicated the non-uniform distribution of polymorphism over six regions within each receptor could evidence their different functions.

### Multiplicity of NF docking poses

In order to model NFR5–NF–K1 complexes, we first obtained the docked structure of NFR5–NF complexes. The protein-ligand docking was performed for the dominant haplotype of the NFR5 receptor and 10 generated conformations of *R. leguminosarum* bv. *viciae* NF. We chose six regions for docking: three LysM domains and three grooves between LysM domains. Within each of the regions we obtained different variants of docking, however, some of them were similar to each other. For further analysis, we selected NF poses following two strategies. Under the first strategy (called the “a priori” strategy based on the published data) we took ligand poses within the shallow groove in the LysM2 domain (4 poses total)—this shallow groove was previously shown to bind chitin in homologous receptors. Under the second strategy (the “energy” strategy based on the energy parameters), we selected top 5 dock poses with low values of MM-GBSA within each of six regions for further analysis (Supplementary Figure S3A). Thus we separately analyzed two sets of dock poses with no common variants, with 4 and 30 variants, respectively.

### Protein–protein docking

We performed protein–protein docking for the major haplotypes of NFR5 and K1 receptors in the Schrödinger BioLuminate module. The 30 output complexes passed the first manual selection step with respect to an adequate orientation of complexes to a possible cell membrane. If the putative location of the transmembrane domain of one subunit overlapped with another subunit, then the complex was discarded. Only 15 heterodimers were selected for further analysis.

### Reduction of possible NF poses

Prior to the analysis of possible ligand dock poses, we defined two important thresholds. The first threshold was based on the mean distance between components in a receptor-ligand complex, while the second threshold depended on the minimum distance between the components (see section Materials and Methods). To estimate the thresholds, we measured these distances between NF and the NFR5 receptor across 34 dock poses (4 “a priori” strategy-based and 30 “energy-efficient” strategy-based). The highest mean distance between NF and NFR5 was 4.03 Å, the lowest minimum distance was 1.52 Å. Based on these values we defined two soft thresholds-−5 Å (ceiling rounding) and 1 Å (floor rounding) for the mean and the minimum distance between components in a receptor-ligand complex, respectively. The thresholds do not relate to a covalent bond length or any other physical/chemical constants. The directions of rounding are taken to extend the initial set of possible configurations of NFR5–NF–K1 complexes.

To reduce the set of possible ligand dock poses, we employed the hypothesis that the NF interacts with both of the receptors, NFR5 and K1. We first aligned each NFR5–NF complex with each NFR5–K1 complex by the common part–NFR5. As NF and K1 were bound to the NFR5 receptor independently, these two molecules in the resultant NFR5–NF–K1 complexes were overlapped. We took only those NFR5–NF–K1 complexes that satisfied two constraints: (1) the number of overlapped atoms between NF and K1 was not higher than 10 with respect to the 1 Å threshold; (2) the mean distance between NF and K1 was not higher than 5 Å (Supplementary Figure S3B).

**Figure 5:**
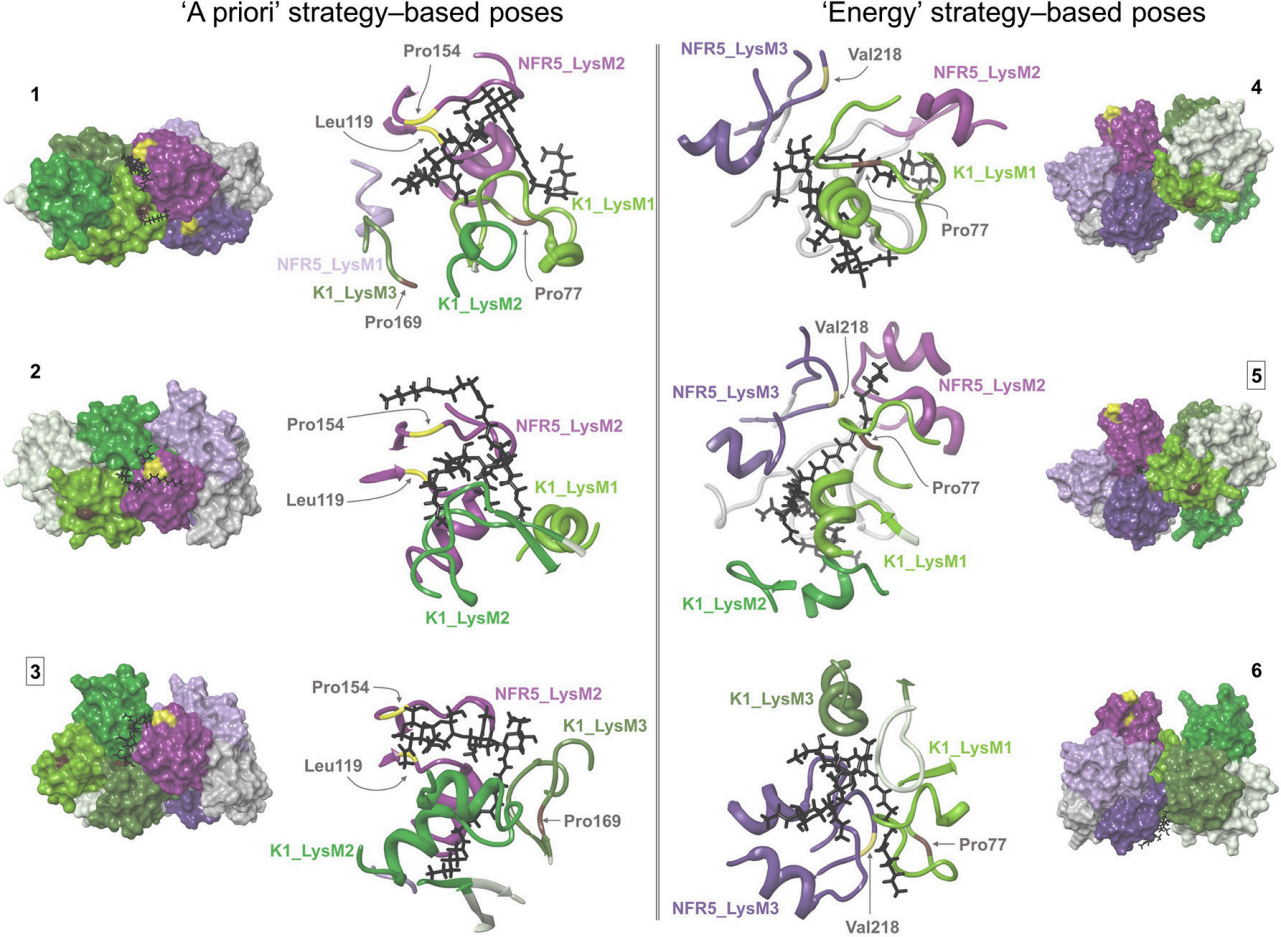
Six NFR5–NF–K1 complexes obtained after the merge of NFR5–K1 and NFR5–NF docking results. Two complexes that were stable after molecular dynamics simulations are marked with squared numbers. **(1–3)** NFR5–NF complexes, which were selected by the “a priori” strategy, when the backbone of Nod factor is located within the shallow groove on the LysM2 domain of NFR5. **(4–6)** NFR5–NF complexes which were selected by the “energy” strategy (lowest MM-GBSA energy).

For the ligand poses selected by the “a priori” strategy, we found three NFR5–NF–K1 complexes satisfied the constraints. For the ligand poses selected by “energy” strategy, we found six NFR5–NF–K1 complexes satisfied the constraints. Within the last six NFR5–NF–K1 complexes we indicated three clusters. Each cluster contained complexes with equal NFR5–K1 heterodimer orientation and almost similar NF orientation (Supplementary Text 1, Supplementary Figure S3C). Within each cluster, we selected one structure with the lowest energy (Supplementary Figure S3A). As a result, we obtained three NFR5–NF–K1 complexes following each strategy, i.e., six complexes in total (Figure 5). Amino acid positions in NFR5 and K1 receptors that are homologous to residues in other LysM-RLKs essential for NF recognition are marked in Figure 5 and described in Supplementary Text 2.

**Figure 6:**
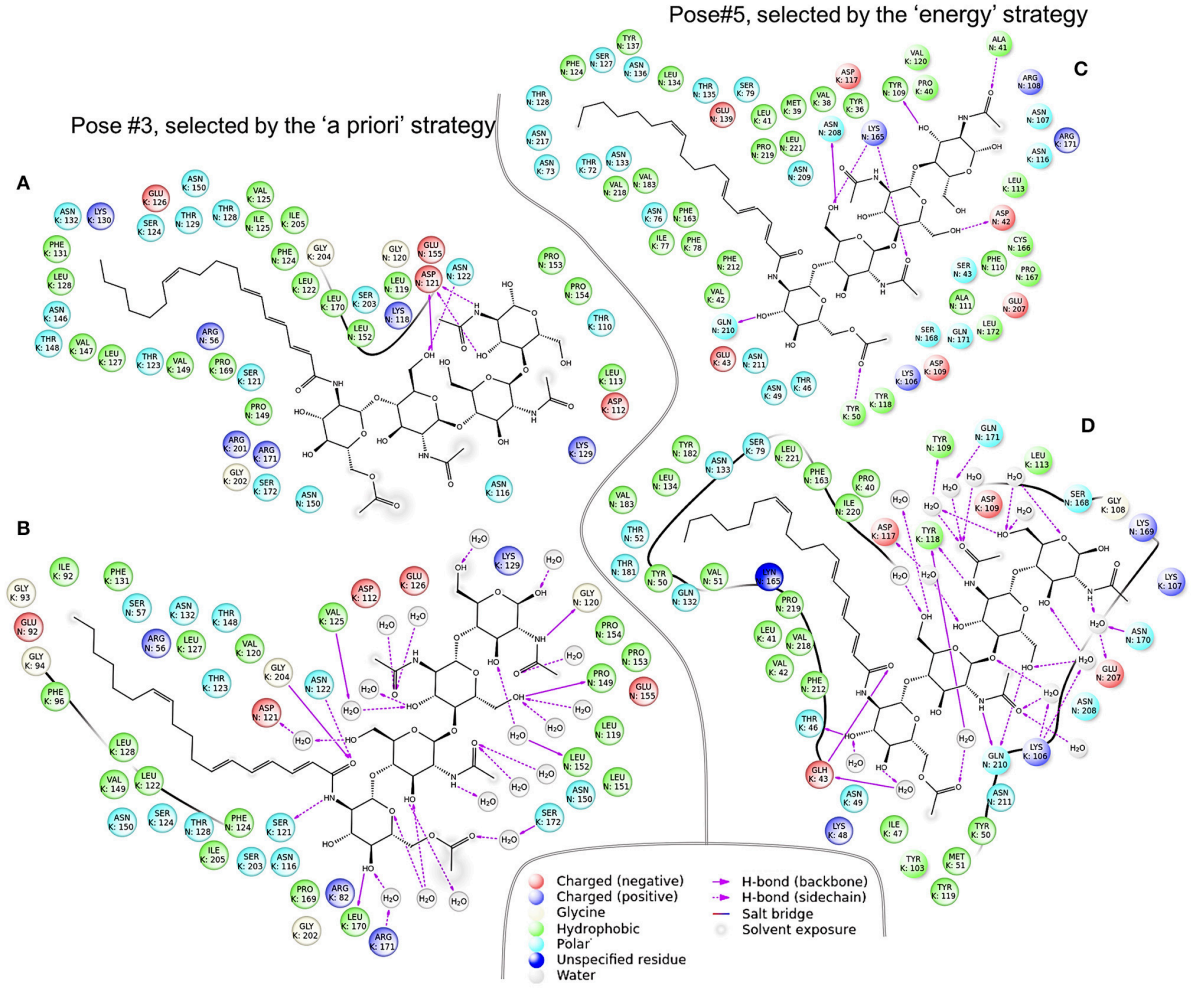
Two NFR5–NF–K1 complexes before **(A,C)** and after **(B,D)** molecular dynamics simulations. Panel **(A,B)** complexes represent Pose#3 configuration, which was selected by the “a priori” strategy. **(C,D)** Represent Pose#5 configuration, which was selected by the “energy” strategy.

For each of the six complexes, we performed the molecular dynamics simulation for 100 ns and extracted an optimal complex by clustering of the frames in the simulation trajectory. Two out of the six optimal complexes were similar to the respective initial complexes and were denoted as stable: Pose#3 and Pose#5 (Figure 6). Unstable configurations had a free NF tail, not grasped in the cavity of the NFR5–K1 contact zone. Both stable configurations were of the similar “sandwich-like” one. To be specific, a LysM domain of one receptor was bound to the groove between LysM2 and LysM3 domains of another receptor (the largest of three grooves). The NF was located in the cavity formed by the contact zone of NFR5 and K1 molecules.

### Polymorphism in the contact zone

The contact zone in the Pose#3 complex was formed mostly by the NFR5_LysM2 and K1_Groove23 regions. These two regions were the most conservative in both receptors (Figure 4C). The contact zone in the Pose#5 complex was formed mostly by the NFR5_Groove23 and K1_LysM1 regions, which were both highly variable (Figure 4D). These observations indicated a congruence in the diversity level between putative interacting parts of NFR5 and K1 receptors, and did not reject the possibility that both of the complexes displayed mutual polymorphism in the contact zone.

**Figure 7:**
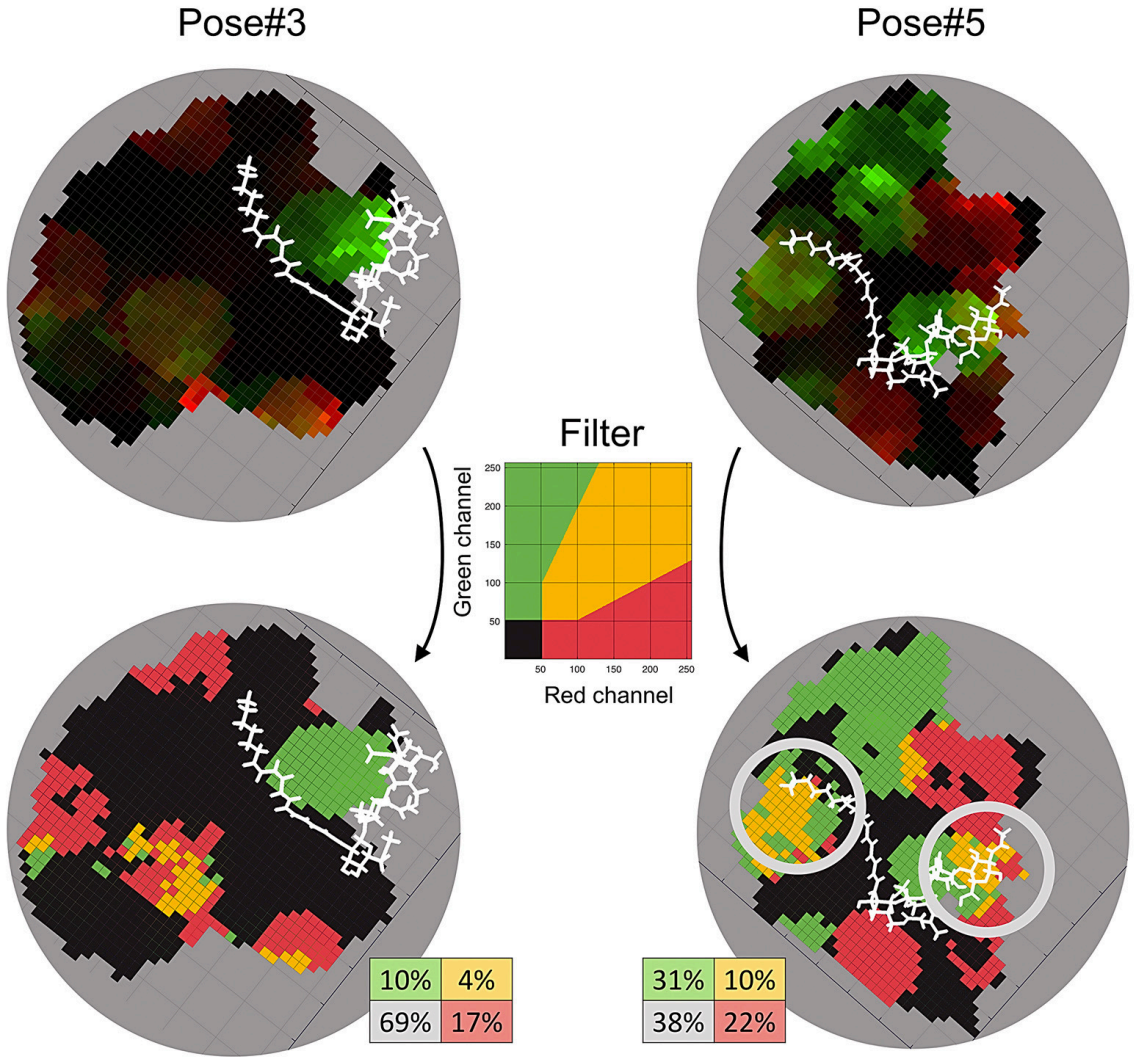
RG-plots for contact zones of Pose#3 and Pose#5 complexes. Two top plots represent initial RG-plots when the two bottom plots represent RG-plots after the filtration. The filter is shown in the middle. The colors mean the following: black—mutually conservative zones; yellow—mutually polymorphic zone; red—zones, where polymorphism from the NFR5 side is more than twice as large as the same from K1 side; green—zones, where polymorphism from the K1 side is more than twice as large as the same from NFR5 side.

We applied the developed Sliding cylinder technique to analyse the mutual polymorphism in two complexes—Pose#3 and Pose#5. Considering the intensity of NFR5 and K1 polymorphisms in the contact zones by red (R) and green (G) channels respectively, we obtained RG-plots for both complexes (Figure 7). We then applied the filter to distinguish four areas: black-colored area showed mutual conservatism; yellow-colored area showed the mutual polymorphism, a zone of a comparable level of polymorphism from NFR5 and K1 sides; and red- or green-colored areas showed the prevailing polymorphism of one receptor, NKFR or K1, respectively. Yellow and black areas were of special interest as they reflect mutual effects (mutual polymorphism and conservatism). The contact zone of the Pose#3 complex contained a large black area (69%), where both receptors displayed no polymorphism. Yellow and black areas of the Pose#3 complex were in total 74% of the contact zone. The Pose#5 complex had around half of the contact zone (48%) colored by either yellow or black. An example of pN/pS analysis in the contact zone of Pose#3 and Pose#5 complexes via RG-plots is shown in Supplementary Figure S4.

In both of the Pose#3 and Pose#5 complexes, the NFs were mostly located in the areas of the mutual conservatism (black areas). We also analyzed the co-localisation of yellow regions on RG-plots with possible variation in NF decorations. A natural population of *R. leguminosarum* bv. *viciae*, as well as individual strains, synthesizes the mixture of NFs with small variations in two parts: (1) the tip of the NF fatty acid tail and (2) the reducing end of the Nod factor backbone. Both the first and the second parts of the NF were overlapped with yellow areas in contact zones of Pose#5 (obtained by the “energy” strategy; Figure 7). The same trends in the Pose#3 complex (obtained by the “a priori” strategy) were not detected.

## Discussion

The key step in the formation of the rhizobia-legume symbiosis is the dialogue between partners through Nod factors (NFs, signaling molecules) from the rhizobia side and the system of receptors from the plant side that specifically recognizes the NF decorations. In spite of the fact that putative NF receptors were discovered more than 10 years ago, the direct interaction of NFs with any of the receptors was demonstrated in only a few studies (Broghammer et al., 2012; Sørensen et al., 2014). We supposed that the reason that blocks the experimental detection of this direct interaction concerns the hypothesis of a heterodimer receptor for NF. Here, we tried to shed the light on the hidden interaction between the NF produced by *R. leguminosarum* bv. *viciae* and its two putative receptors—NFR5 and K1 of *Vicia sativa*—and integrated the population polymorphism of these NFR5 and K1 receptors into the pipeline of modeling the structure of NFR5–NF–K1 complex.

The analysis of the population polymorphism data guided several steps of our study. First, employing the population polymorphism data of *NFR5* and *K1* genes we demonstrated the significant congruence (*p* < 0.05) between *NFR5* and *K1* gene trees that allows us to select corresponding NFR5 and K1 alleles for modeling. The observed congruence suggested the coevolution of *NFR5* and *K1* genes, which was likely caused by the interaction between products of these genes (especially considering the fact that *V. sativa* is a cross-pollinated plant). The analysis of tanglegrams between *NFR5* and *K1* gene trees demonstrated that the dominant haplotypes of these genes corresponded to each other, therefore we took them for further modeling.

We carried out the NFR5–NF docking within six regions (three LysM domains and three grooves between the domains) and selected two sets of NF dock poses based on “a priori” and “energy” strategies. The first set contained poses similar to those previously mentioned in the literature, while the second set contained poses with low MM-GBSA energy scores. Both sets totalled 34 poses overall and this large amount of possible alternative NF docking poses could be explained in two ways. First, this multiplicity of poses probably related to the wide spectrum of functions carried out by evolutionary relatives of studied receptors—homologous systems for chitin perception in plant-parasite interactions and for Myc factor recognition in arbuscular mycorrhizal symbioses, or sometimes for interaction with both types of signals (Miyata et al., 2014). Thus, the observed multiplicity probably included some non-actual variants for rhizobia-legume symbiosis but appropriate poses for binding (lipo-) chitooligosacharide signaling molecules in other plant-microbe interactions. Second, the multiplicity of NF dock poses contains the actual one, but it can be determined only when the position of the second subunit of the heterodimer is presented.

To reduce the number of NFR5–NF variants obtained, we performed NFR5–K1 docking and combined the heterodimers with the 34 NFR5–NF variants. We obtained six NFR5–NF–K1 complexes where the signaling molecule was partially or completely located within the cavity formed by the contact zone of the NFR5 and K1 receptors. After 100 ns dynamics simulations, two out of the six tested complexes maintained the topology. These result demonstrated the principled possibility of NFR5–NF–K1 complexes where the NF binding site was formed by both of the receptor subunits.

One of the stable NFR5–NF–K1 configurations, Pose#3, corresponded to the set of NFR5–NF dock poses selected by the “a priori” strategy, while the other configuration, Pose#5, corresponded to the set selected under the “energy” strategy. We observed the comparable level of population polymorphism in contacted parts of NFR5 and K1 in both NFR5–NF–K1 configurations: the contact zone of Pose#3 complex was formed by two the least polymorphic regions from NFR5 and K1 receptors, while the contact zone of the Pose#5 complex involved two highly variable regions. This result supported the possible presence of the mutual polymorphism/conservatism in the contact zones of both complexes. We then applied the deeper analysis of population polymorphism within the contact zone by the Sliding cylinder technique. This method generates RG-plots, where yellow-colored areas indicate zones of mutual polymorphism, black-colored areas show zones of the mutual conservatism and other areas display a non-comparative level of population polymorphism from NFR5 and K1 sides. We also made projections of the NF to these plots (Figure 7).

We found that the contact zone in the Pose#3 (selected by the “a priori” strategy) was mostly mutually conservative and black and yellow zones covered, in total, 74% of the contact zone. We hypothesized that zones of the mutual conservatism could participate in a stabilization of the NFR5–K1 structure and also in a perception of conservative parts of NFs. The contact zone of the Pose#5 configuration (selected by the “energy” strategy) contained 48% of mutually polymorphic/conservative zones. However, two large yellow-colored zones of mutual polymorphism were overlapped with the NF variable parts in a natural population of *R. leguminosarum* bv.*viciae*. Based on the results, we assumed that zones of mutual polymorphism in the NFR5–K1 contact zone can be an indicator of binding with variable NF parts. Population polymorphism within red and green zones probably does not affect interactions in the NFR5–NF–K1 complex. In the recent study, we assumed that the natural variation in NF decorations could be responsible for the Evolutionary Moulding—the matching of population diversities of rhizobial nodA and legume NFR5 genes. The demonstrated here mutual polymorphism in the contact zone of one NFR5–NF–K1 complex (Pose#5) supports our assumption, as the zones of mutual polymorphism overlapped with a variable part of NF, which was proposed as a mediator in the Evolutionary Moulding.

**Figure 8:**
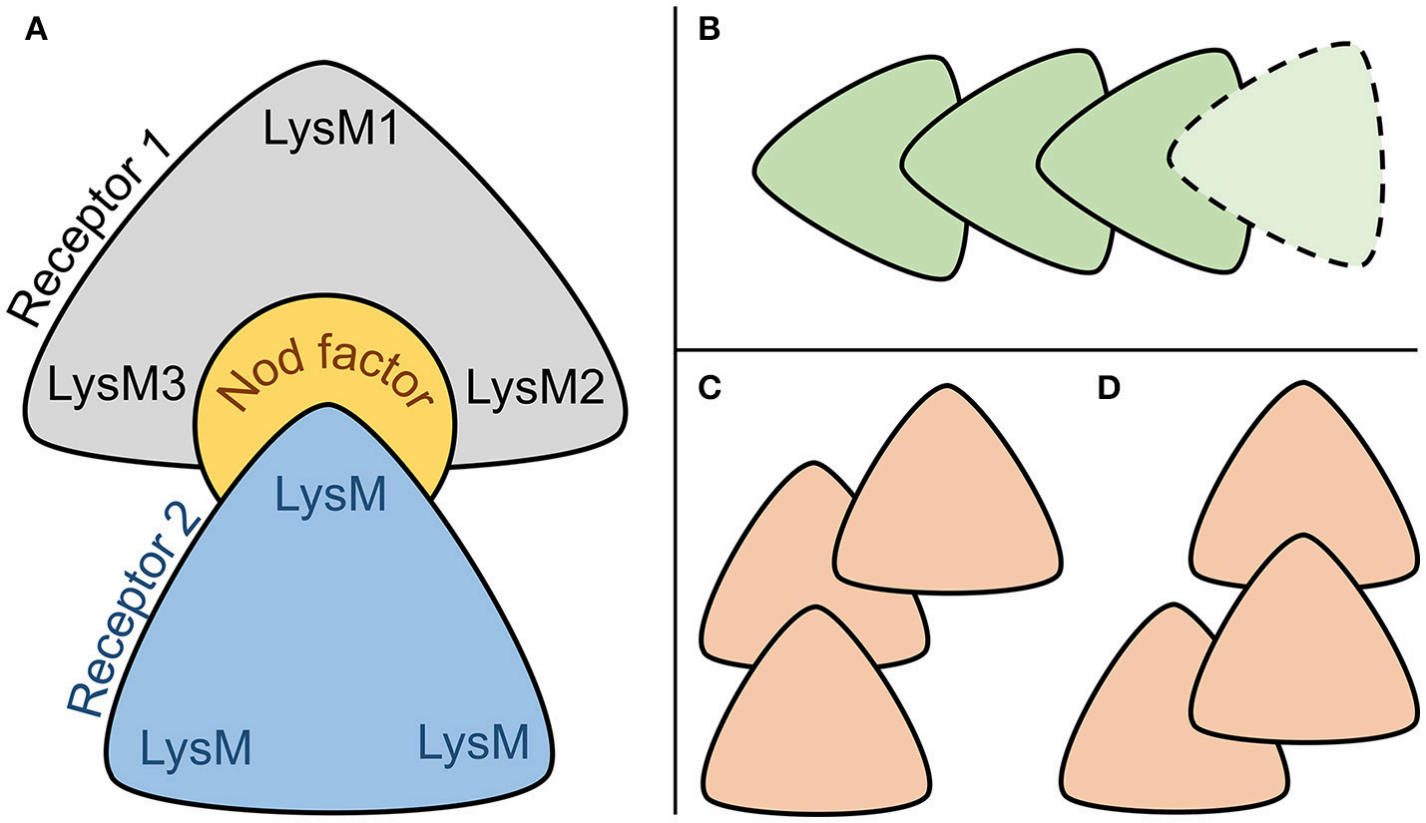
**(A)** Common sandwich-like configuration of two stable NFR5–NF–K1 complexes. **(B–D)** Configurations of interacting LysM-RLKs which are possible under the hypothesized principal of heteromerization.

Both stable complexes, Pose#3 and Pose#5, were represented by the same “sandwich-like” configuration of the heterodimer subunits (Figure 8A). We proposed that this “sandwich-like” principle could be common for heteromerization and homomerization of LysM-RLKs. According to this principle, we assumed several topologies for heterotrimeric complexes proposed recently (Figures 8B–D) (Zipfel and Oldroyd, 2017). Moreover, we do not exclude the possibility of a receptor complex consisting of four or more LysM-RLKs formed with the principal (Figure 8B).

This study is the first in which the possible 3D configuration of the two-receptor system was demonstrated, the common principle of heteromerization of LysM-RLKs was proposed and the population polymorphism of receptors was analyzed in the context of NFR5–NF–K1 complexes. We showed that the mutual polymorphism is an important feature in understanding not only the configuration of the NFR5–NF–K1 complex but also the functional role of the natural polymorphism of receptors within a *V. sativa* population.

## Author contributions

AI: performed all calculations and molecular modeling, developed Sliding cylinder technique and wrote the article with contributions from coauthors; YP: performed the critical revision of the molecular modeling, contributed to writing the article; EC: constructed primers for sequencing and performed the wet lab work; EA: developed the conception or design of the work, contributed to writing the article.

### Conflict of interest statement

The authors declare that the research was conducted in the absence of any commercial or financial relationships that could be construed as a potential conflict of interest.
