# Supplementary material for: Structural Insight Into the Role of Mutual Polymorphism and Conservatism in the Contact Zone of the NFR5–K1 Heterodimer With the Nod Factor
Source: Front Plant Sci. 2018 Apr 11;9:344. doi: 10.3389/fpls.2018.00344 (PMC5909492; doi:10.3389/fpls.2018.00344)
Supplement: Supplementary file 1 [file Presentation1.PDF]

Supplementary File

Text 1

Protein-Ligand Contact Zone Area

The area of a contact zone between a protein and a ligand in a docked complex cannot be directly calculated by Schrödinger tools. Therefore, we determined the area of the contact zone through areas of three surfaces: (1) the whole surface of the docked configuration of ligand - S_{lig}, (2) the whole surface of the protein - S_{prot}, (3) the whole surface of the docked complex $S_{complex}$. The area of the contact zone was calculated as follows:

$$S_{contact} = \frac{1}{2}(S_{prot} + S_{lig} - S_{complex})$$

Text 2

Analysis of the residuals in 5Å area around the Nod factor in NFR5-K1-NF complexes

In both of the obtained NFR5-K1-NF complexes, residuals essential for Nod factor perception were found. To be specific, Pose#3 complex had within 5Å area VsNFR5_Leu119 (homologous to LjNFR5_Leu118), VsNFR5_Pro154 (homologous to MtNFP_Leu154) and VsK1_Pro169 (homologous to PsK1_Pro169). Pose#5 complex had within 5Å area VsNFR5_Val218 (homologous to MtLYR3_Tyr228), VsK1_Ile77 (homologous to PsSym37_Leu77). After the molecular dynamics all of the mentioned residuals, except VsK1_Ile77, remained in the 5Å area (Fig. 5). The presence of the essential positions near the Nod factor in both complexes only increased the likelihood of their structure.

Figures

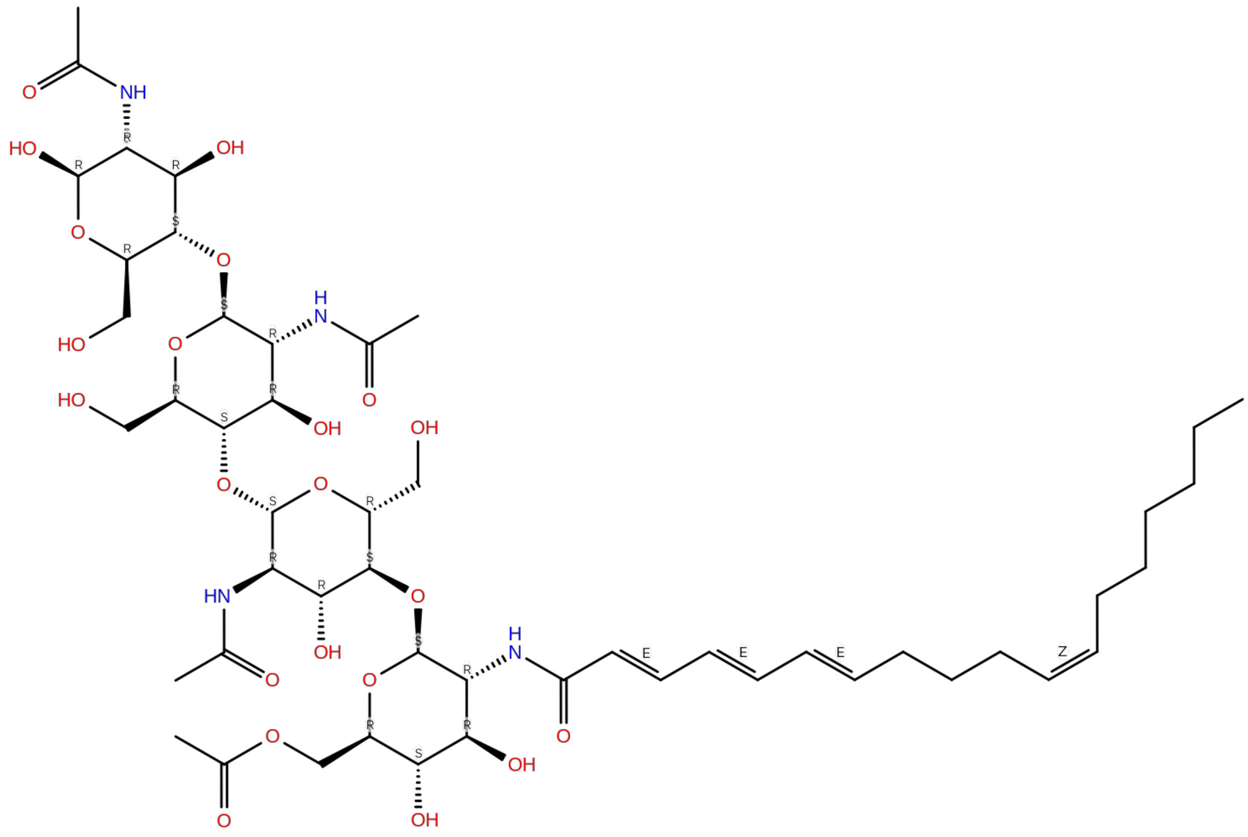

Supplementary Fig. S1. Two dimensional representation of Nod factor.

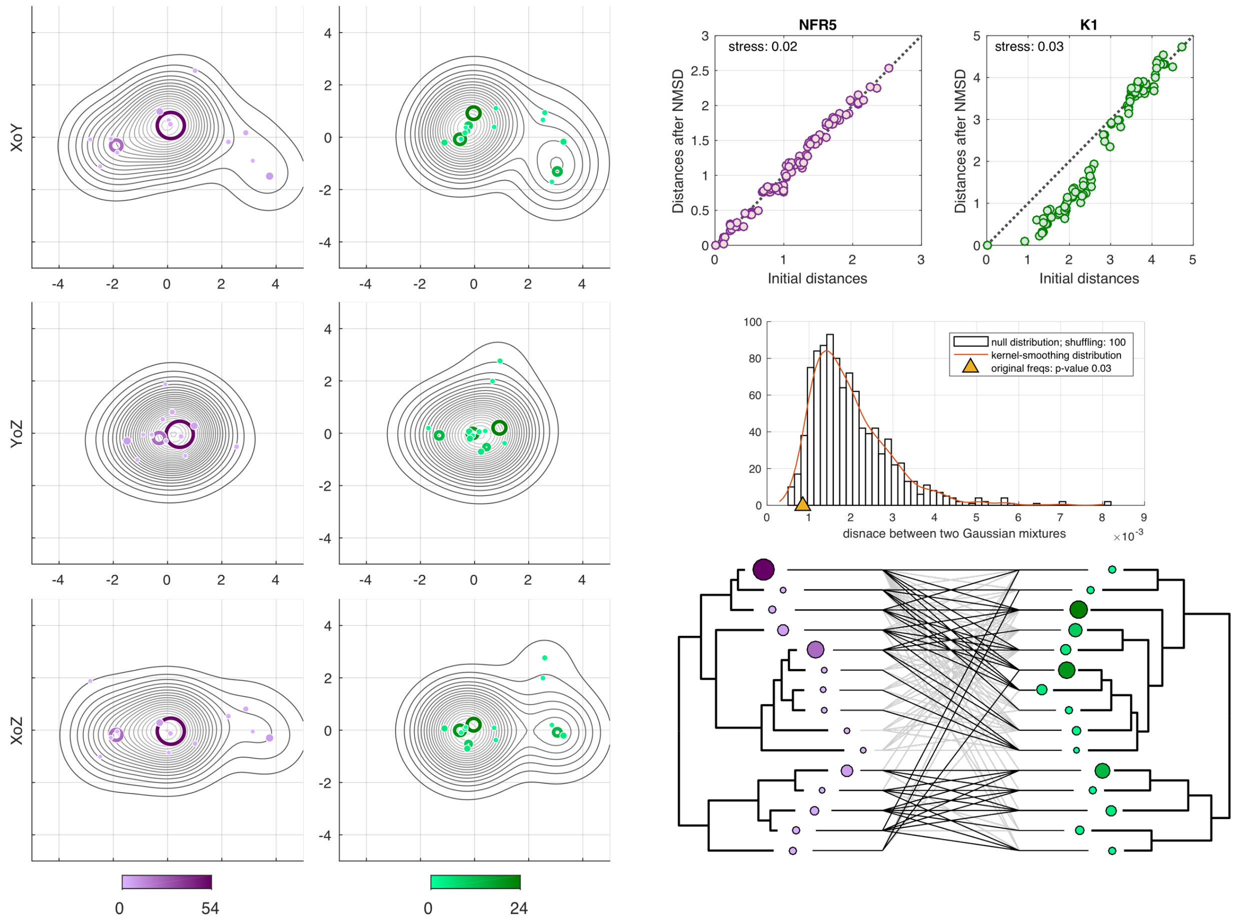

Supplementary Fig. S2. The full results of congruence analysis of *NFR5* and *K1* gene trees: Projections of GMMs to tree coordinate planes, the accuracy of MDS, the p-value of ΔG value and the final congruence between gene trees.

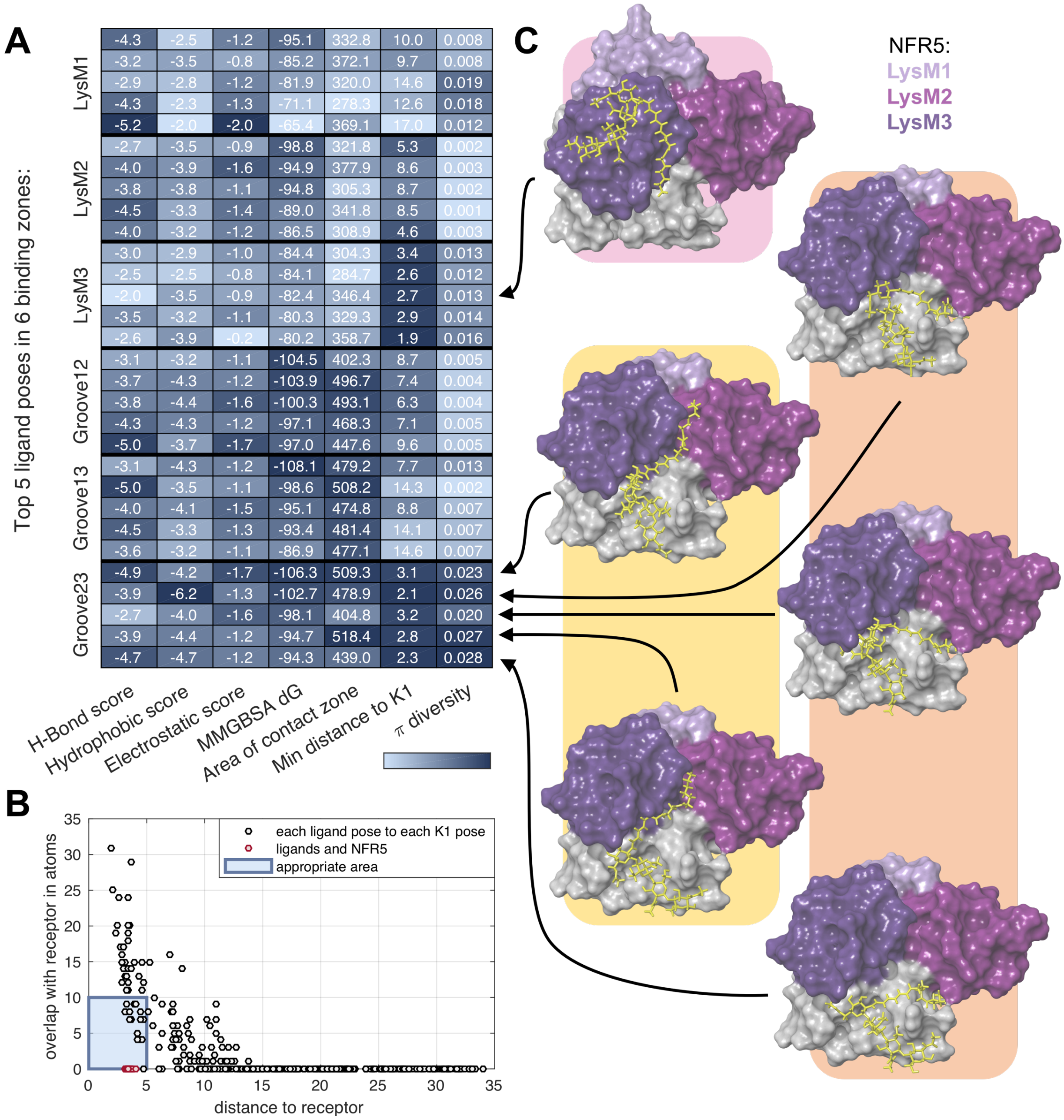

Supplementary Fig. S3. (A) Summary statistics for top 5 Nod factor dock poses within each of six regions. (B) The characteristics of all NFR5-K1-NF configurations: mean distance between NF and K1 (X-axis), number of overlapped atoms of NF with K1 (Y-axis). (C) Six NF poses selected by 'energy' strategy and a close distance to K1 formed 3 clusters.

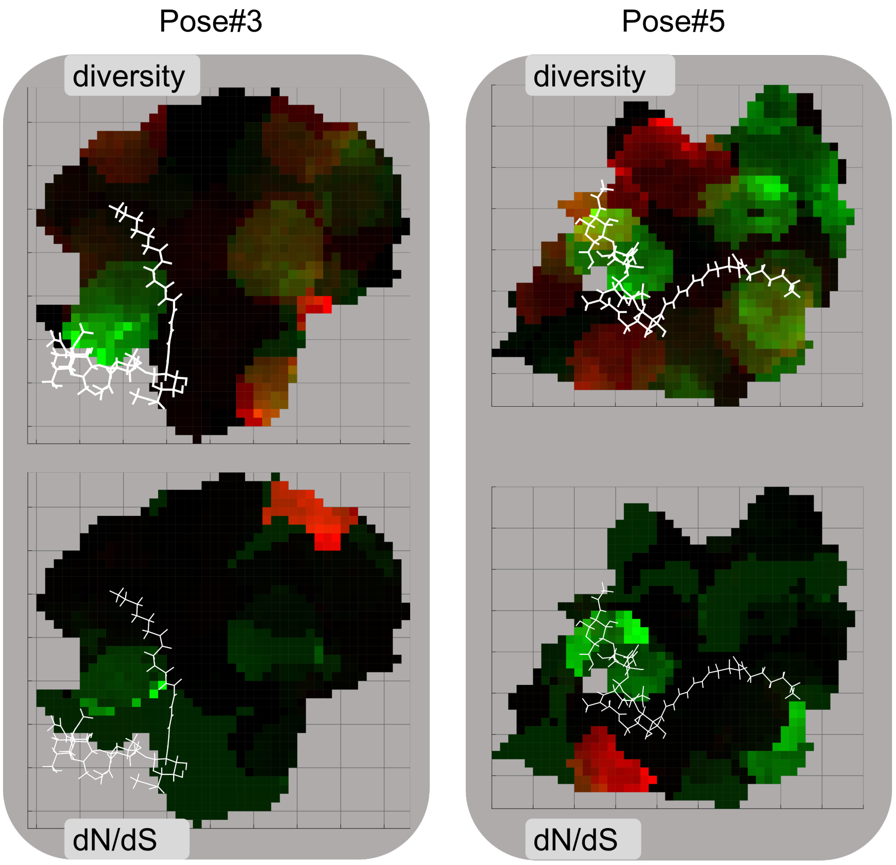

Supplementary Fig. S4. Mutual polymorphism and pN/pS statistics within the contact zone of Pose#3 and Pose#5 complexes. Red colour denotes the polymorphism in NFR5, Green colour denotes the in K1

Tables

Supplementary Table S1. Residuals of NFR5 in six putative dock regions determining the centroids of the Glide grid

Putative pose	IDs of residuals of NFR5
LysM1	53:99
LysM2	115:161
LysM3	182:223
Groove12	129 130 131 132 133 117 116 115 52 56 57 58 62 65 66 67
Groove13	53 93 94 53 95 97 187 193 196 197 224 226 227 229 79 80 82 83 178 225
Groove23	41, 42, 48, 105, 106, 109, 110, 111, 112, 133, 134, 135, 136, 139, 143, 163, 165, 166, 167, 168, 181, 183, 208, 209, 210, 212, 217, 218, 219, 221
